# A Randomized Cross-over Air Filtration Intervention Trial for Reducing Cardiovascular Health Risks in Residents of Public Housing near a Highway

**DOI:** 10.3390/ijerph120707814

**Published:** 2015-07-10

**Authors:** Luz T. Padró-Martínez, Emmanuel Owusu, Ellen Reisner, Wig Zamore, Matthew C. Simon, Mkaya Mwamburi, Carrie A. Brown, Mei Chung, Doug Brugge, John L. Durant

**Affiliations:** 1 Department of Civil and Environmental Engineering, Tufts University, Medford, MA 02155, USA; E-Mails: lpadro@gatech.edu (L.T.P.-M.); matthew.simon@tufts.edu (M.C.S.); 2 City of Somerville, Housing Division, Somerville, MA 02145, USA; 3 Somerville Transportation Equity Partnership, Somerville, MA 02145, USA; E-Mails: reisnere51@gmail.com (E.R.); wigzamore@gmail.com (W.Z.); 4 Department of Public Health and Community Medicine, Tufts University Boston, MA 02111, USA; E-Mails: mkaya.mwamburi@tufts.edu (M.M.); mei_chun.chung@tufts.edu (M.C.); dbrugge@aol.com (D.B.); 5 Jean Mayer USDA Human Nutrition Research Center, Tufts University, Boston, MA 02111, USA; E-Mail: carrie.brown@tufts.edu

**Keywords:** in-home filtration, HEPA, highway, ultrafine particles, biomarkers, inflammation

## Abstract

Exposure to traffic-generated ultrafine particles (UFP; particles <100 nm) is likely a risk factor for cardiovascular disease. We conducted a trial of high-efficiency particulate arrestance (HEPA) filtration in public housing near a highway. Twenty residents in 19 apartments living <200 m from the highway participated in a randomized, double-blind crossover trial. A HEPA filter unit and a particle counter (measuring particle number concentration (PNC), a proxy for UFP) were installed in living rooms. Participants were exposed to filtered air for 21 days and unfiltered air for 21 days. Blood samples were collected and blood pressure measured at days 0, 21 and 42 after a 12-hour fasting period. Plasma was analyzed for high sensitivity C-reactive protein (hsCRP), interleukin-6 (IL-6), tumor necrosis factor alpha-receptor II (TNF-RII) and fibrinogen. PNC reductions ranging from 21% to 68% were recorded in 15 of the apartments. We observed no significant differences in blood pressure or three of the four biomarkers (hsCRP, fibrinogen, and TNF-RII) measured in participants after 21-day exposure to HEPA-filtered air compared to measurements after 21-day exposure to sham-filtered air. In contrast, IL-6 concentrations were significantly higher following HEPA filtration (0.668 pg/mL; CI = 0.465–0.959) compared to sham filtration. Likewise, PNC adjusted for time activity were associated with increasing IL-6 in 14- and 21-day moving averages, and PNC was associated with decreasing blood pressure in Lags 0, 1 and 2, and in a 3-day moving average. These negative associations were unexpected and could be due to a combination of factors including exposure misclassification, unsuccessful randomization (*i.e.*, IL-6 and use of anti-inflammatory medicines), or uncontrolled confounding. Studies with greater reduction in UFP levels and larger sample sizes are needed. There also needs to be more complete assessment of resident time activity and of outdoor *vs.* indoor source contributions to UFP exposure. HEPA filtration remains a promising, but not fully realized intervention.

## 1. Introduction

Ultrafine particles (UFP; diameter ≤0.1 μm) in urban air derive from many anthropogenic sources—e.g., combustion of biogenic and fossil fuels, condensation of organic vapors, tire wear, brake wear, aerosol sprays, cigarette smoking, and cooking [1,2,3,4]. While the health impacts of exposure to UFP are less well understood compared to exposure to larger particles (e.g., PM_2.5_, diameter ≤ 2.5 μm [5,6,7,8]), there is concern that UFP could be particularly toxic. Due to their small size, they can penetrate deeply into the lungs [9] and then into the blood stream [10]. Also, compared to larger particles they contain relatively high concentrations of toxic chemicals such as polycyclic aromatic hydrocarbons and reactive metal species [11,12]. Recent studies have found associations of UFP acute exposure with blood pressure [13,14,15] and blood markers of inflammation and coagulation [6,16,17].

Motor vehicle exhaust is a significant source of UFP and as a result UFP concentrations are generally elevated near major roadways [18,19,20,21,22]. Studies have also shown that people living within 200–300 m of major roadways are at increased risk of cardiovascular disease [23,24,25], leading to the hypothesis that exposure to near-highway air pollutants (including UFP) may be responsible. Even though people spend most of their time indoors [26], exposure to UFP of outdoor origin can occur indoors due to infiltration into buildings and homes. Infiltration is most significantly impacted by window opening and air conditioner use [27,28]. While indoor sources of UFP such as smoking, cooking, cleaning (vacuuming, sweeping, dusting, and use of aerosol cleaners), and resuspension of particles deposited on indoor surfaces contribute significantly to personal exposures [3,29,30], UFP of outdoor origin can also represent a significant fraction of indoor personal exposures [27,28,31]. 

In an effort to reduce indoor exposures to airborne particles, several studies have investigated the use of high-efficiency particulate arrestance (HEPA) filters. HEPA filtration has been shown to reduce particulate matter (PM) concentrations as much as 70%–80% (depending on particle size) in field applications [4,32]. Furthermore, in-home HEPA filtration has been found to improve asthma in children [33,34] and reduce some, but not all, markers of cardiovascular risk in adults [35,36]. With the exception of Bräuner *et al*. [36], who performed a short term air-filtration study in a population living near a roadway (>10,000 vehicles per day) in Copenhagen, to date little work has been done to measure the health benefits of HEPA filtration for people living near busy roadways [37].

Our goal was to conduct a public health intervention study aimed at improving indoor air quality that could have a positive effect on markers of cardiovascular disease risk. The population consisted of residents of public housing located within 200 m of Interstate 93 in Somerville, Massachusetts, USA (>150,000 vehicles per day [38]). Our specific objectives were to: (1) operate HEPA filtration units in the homes of 20 participants for six weeks and measure UFP concentrations in HEPA-filtered and unfiltered air; (2) measure blood pressure and blood biomarkers in participants before and after exposure to HEPA-filtered and unfiltered air; and (3) determine whether differences in UFP concentrations were associated with the biological measurements. The blood biomarkers included markers of inflammation (*i.e.*, high sensitivity C-reactive protein (hsCRP), interleukin-6 (IL-6), and tumor necrosis factor alpha-receptor II (TNF-RII)) and coagulation (*i.e.*, IL-6 and fibrinogen). hsCRP and fibrinogen are associated with cardiovascular disease risk [39,40], and hsCRP, IL-6, and TNF-RII are reported to be associated with exposure to ultrafine particles [6,16,41]. The study was a Community Based Participatory Research (CBPR) project involving the City of Somerville (Housing Division), Somerville Transportation Equity Partnership (STEP), and the Schools of Medicine and Engineering at Tufts University [42]. Recruitment efforts were informed by the Community Assessment of Freeway Exposure and Health (CAFEH) study [43], which increased awareness of traffic-related air pollution problems in our target community and helped to promote the study as a response to the concerns of residents.

## 2. Experimental Section 

### 2.1. Study Location and Design

**Figure 1:**
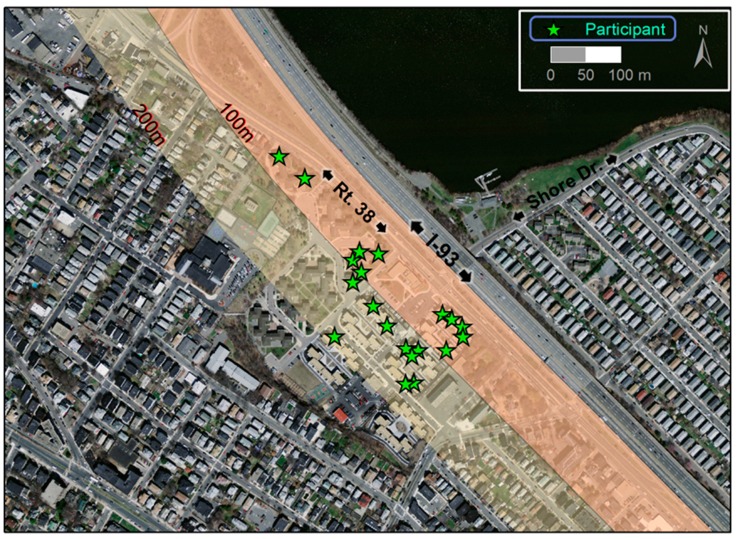
Map of study area and locations of participant apartments. Orthophoto was downloaded from MassGIS [44].

The study was conducted in 20 apartments (21 participants) in the Mystic View and Mystic River housing developments in Somerville. One apartment was excluded from the analysis due to a problem with the air monitoring equipment; thus, our final data set included 19 apartments and 20 participants. The two developments are adjacent to one another as well as to I-93 and Massachusetts State Highway 38 (Rt-38; >30,000 vehicles per day [38]; Figure 1). I-93 rises to as high as 6-m-above grade next to the developments and is filled underneath except for the underpass at Shore Drive; Rt-38 runs at grade parallel to I-93. Participants were at least 40 years old, did not smoke or allow smoking inside their homes, and their apartments were within 200 m of I-93. Recruitment was in three languages: English, Haitian Creole and Spanish. Details of recruitment and our interaction with the participants are described elsewhere [42].

The study design was a randomized, double-blind crossover trial with the goal of having 50% of participants start with HEPA filtration and 50% start with sham filtration. To facilitate scheduling, pairs of participants were studied in parallel with one participant starting with HEPA and the other with sham filtration with assignment randomized. The purpose of randomization was to minimize the effects of temporal variation in PNC and blood biomarker concentrations on the results. Each participant was exposed to HEPA-filtered air for 21 days and unfiltered (sham) air for 21 days. Regardless of which filter was in use (HEPA or sham), the sound and appearance of the equipment was the same; thus, participants did not know which filter was in use. Because each participant was exposed to both filtered and unfiltered air, they served as their own control. 

Associations of PNC with CRP and IL-6 have been found for periods from as little as 1 to as many as 28 days in natural exposure [11,16] and controlled exposure studies [17]. We used a 21-day exposure period because it allowed for testing exposure times toward the longer end of what is reported in the literature while also encompassing shorter time frames. In general, longer exposures to air pollution are associated with greater biological effects than are shorter exposures. For blood pressure we focused on shorter time frames, consistent with other studies that have found associations with air pollution in hours to two days [13,14,15]. 

All participants signed an informed consent form and completed a survey containing questions on demographics, socio-economic status, residential history, time activity, window opening, air conditioner use, potential exposure to combustion sources (at home and work and on highways), and health status. Medication use was obtained by recording the names of medicines found in each apartment. In addition, at the end of the first intervention period and at the end of the study (t = 21 and 42 days, respectively), participants completed supplemental surveys to obtain information on time activity and health status during the preceding week. The time-activity questions obtained hour-by-hour estimates of time spent in five microenvironments—inside home, outside home, school/work, highway travel, and other—on week days and weekend days for unemployed participants and work days and non-work days for employed participants. The consent forms and surveys were forward and back translated and the surveys were conducted by independent translators fluent in both English and the second language. Each participant received stipends for the initial survey, each blood draw, and for electricity use. The study was conducted in accordance with the Declaration of Helsinki, and approved by the Tufts University Institutional Review Board (Protocol #1008078). The study was conducted from February 2011 to November 2012.

### 2.2. Air Filtration and PNC Monitoring

Each apartment had a kitchen, dining area, living room, and two or three bedrooms. Window-mounted HEPAiRx air filtration units equipped with user-controlled air heating and cooling elements (Air Innovations, Inc., North Syracuse, NY, USA; http://www.airinnovations.com/) were used in the study because they have been shown to reduce the severity of asthma in children [33]. These units use a MERV 17 filter (rated to remove ≥99.97% of particles ≥0.3 μm in diameter) and can operate at ~10 exchanges/hour in a <28.3 m^3^ (10^3^ ft^3^) room. The units were installed in the living room of each apartment as opposed to the bedroom because most participants indicated they spent more time each day in their living rooms as well as to minimize potential sleep disruption for participants due to instrument noise. To maximize particle removal, the HEPA units were operated at the highest possible fan speed and the side vents were blocked off so that there was no flow of outdoor air through the unit into the apartment. Filters were changed in each apartment after 21 days (HEPA for sham or vice versa). A new HEPA filter (MERV 17) was used in each apartment. The sham filter, a hollow box made from perforated 1-mm-thick sheet metal, was the same size and shape and had the same appearance as the metal frame around the HEPA filters. A sign was placed on the HEPA-unit cover asking participants (in multiple languages) not to tamper with or expose the filter. While we found no evidence that any of the filters had been tampered with, we cannot completely discount the possibility that some participants did not follow our instructions. Nevertheless, failure of blinding of participants to the filtration regime is of less concern in a study such as ours that has objective health attributes, blood biomarkers and blood pressure in our case, which are not under voluntary control of participants.

The number concentration of particles between 7 and 3000 nm in diameter was measured continuously over the 42-day trial in each apartment using a water-based condensation particle counter (CPC; TSI Model 3783). PNC was measured every second and one-minute averages were recorded. With the exception of apartments 3 and 9, the CPCs were installed in the living room along with the HEPA. Due to space limitations in the living rooms of these two apartments the CPC was installed in the dining rooms, which were immediately adjacent to the living rooms. Before the start of the intervention in each apartment, the air flow rate reported by the CPC was checked using a flow meter (TSI Model 4140) (no discrepancies were observed throughout the study), and the CPC vacuum was checked for leaks by placing a polyethersulfone membrane filter (rated at 99.96% removal efficiency for 0.45 µm particles) on the inlet to insure the CPC measured <100 particles per cm^3^. PNC data was collected weekly and checked for CPC temperature and flow errors flagged by the instrument. Data points with errors (typically <1% of all data) were removed from the data set. After each 42-day trial, the flow rate in the CPCs was measured again. Side-by-side comparisons of the CPCs performed in the laboratory resulted in an R^2^ of 0.91 with paired measurements differing by <10%.

Dimensions of the living rooms ranged from 3.1 to 3.9 m in width, from 4.0 to 5.1 m in length, and the ceiling height of all the apartments was 2.4 m. Thus, the total volume of the living rooms was 34.3 to 40.5 m^3^. Each apartment had baseboard heaters (forced hot water) and no centralized air cooling system or forced-air ventilation. During the summer some participants installed window-mounted air conditioners (AC) and free-standing fans or opened windows to help cool their apartments. In apartments that had an AC in the living room, the AC was exchanged for a HEPAiRx unit, which also contained an AC, to comply with housing authority rules requiring at least one living room window be freely accessible as an emergency exit. Participants were asked to keep their windows closed during the study and to use the HEPAiRx to provide cooling. With the exception of stove fans, which were present in all of the apartments and vented outdoors, none of the apartments was equipped with additional air cleaning technology beyond the HEPAiRx filter units. Participants were instructed to run the HEPA unit and CPC at all times (24 hours a day, 7 days a week) during the 42-day study period. We found no evidence that participants turned off the HEPA units, an important consideration in long-term HEPA filtration studies [45]. 

To better understand the factors affecting PNC reductions by HEPA and sham filtration in participant homes, experiments were performed in an unoccupied bedroom in a Somerville apartment that was >1 km from I-93 and was not in the Mystic View or Mystic River Apartment buildings. The bedroom was 27.8 m^3^ (981 ft^3^), furnished with a bed, bedside table, desk and chair, and had hardwood floors but no rugs or carpeting. Prior to the experiments the air vent and gaps around the door were sealed. Particle removal efficiencies were measured for three kinds of sources: (1) no source; (2) a continuous, low concentration source (*i.e.*, bedroom window open 2.8 × 10^3^ cm^2^ throughout the experiment); and (3) a discrete, high concentration source (*i.e.*, a burning candle lit for 45 min and then extinguished). Each experiment was performed in triplicate with a HEPA filter and in triplicate with a sham filter. PNC reductions were calculated using Equation (1):

(1) $$\frac{\left(PNC_{HEPAoff}-PNC_{HEPAon}\right)}{PNC_{HEPAoff}}\;x\;100$$

where *PNC_HEPA off_* was the mean PNC during the 30-min period immediately before the HEPA unit was turned on, and *PNC_HEPA on_* was the mean PNC between 10 and 40 min after the HEPA unit was turned on (0–10 min was excluded because the new baseline was not yet reached). The PNC reduction for the candle experiments was calculated based on the change in PNC after the candle was extinguished with the HEPA running continuously.

### 2.3. Blood Biomarker and Pressure Measurements

Nurses from the Visiting Nurses Association (VNA) of Eastern Massachusetts performed three visits to each participant’s apartment: on day 1 just before HEPA/sham filtration was started, on day 21 1–2 h before the filters were changed, and on day 42 just before the end of the intervention. On the day-1 visit weight and height were recorded using a standard scale (SECA, Model #8761321009) and stadiometer (Model #905055, Shorr Productions LLC, Olney, MD, USA), and blood lipid profile was measured from a finger stick using a CardioChek PA device (Polymer Technology Systems, Inc., Indianapolis, IN, USA). On all three visits diastolic and systolic blood pressure were measured in the right and then left arms of seated participants using an automatic blood pressure machine (Model #HEM711ACN2, Omron Healthcare, Kyoto, Japan). A venous blood sample was collected during each visit and then transported to the Clinical & Epidemiologic Research Laboratory at Boston Children’s Hospital, where it was processed to plasma and stored at minus 80 ºC within 1–3 h of collection. Participants were instructed to fast overnight prior to the blood draws, which occurred between 8 and 10 AM. Samples were assayed in batches using immunoassay kits for hsCRP (SPQ High Sensitivity CRP Reagent Set; DiaSorin, Stillwater, MN, USA), fibrinogen (κ-Assay; Kamiya Biomedical, Seattle, WA, USA), TNF-RII (Quantitative, R&D Systems, Minneapolis, MN, USA) and IL-6 (Quantitative HS, R&D Systems). Quality assurance protocols developed by the lab were used to check the quality of the data. The lab was blinded to the intervention status of the blood samples. 

### 2.4. Data Analysis

For the primary analysis, differences in blood biomarker and blood pressure measurements during exposure to HEPA and sham filtration, independent of filtration order, were assessed with a paired *t*-test. In addition, the blood biomarker data was divided into four groups based on the treatment received (HEPA or sham) and the order participants were exposed to each filter, and then compared using generalized estimating equations (GEE) to account for non-independence of measurements [46]. Blood biomarkers—hsCRP, Fibrinogen, TNF-RII and IL-6—were log transformed (natural log) prior to analysis. Results presented have been back transformed and are reported as percent changes. The GEE analysis was performed using the GENMOD procedure in SAS (v9.3, SAS Institute Inc., Cary, NC, USA) and was checked by a third party check for coding errors (there were none). The least squared means adjusted for baseline are used to report the change in blood biomarker concentrations during each three-week exposure period (either HEPA or sham). A *t*-test showed a significant difference between treatment groups for IL-6 (*p* = 0.05) at baseline, indicating that randomization for IL-6 was not ideal. Accordingly, we also analyzed IL-6 using a repeated measure analysis.

We conducted exploratory tests of the sensitivity of our results to PNC exposure by analyzing PNC at different exposure times prior to the collection of blood samples and blood pressure measurements. Exposure-time windows included Lag0, Lag1, and Lag2 (*i.e.*, the median of all PNC measurements 0–24, 24–48 and 48–72 h before the biological measurements, respectively), and moving average (MA)3, MA14, and MA21 (*i.e.*, the median of all PNC measurements from 0 to 3, 0 to 14, and 0 to 21 days prior to the biological measurements, respectively).

Because participants did not spend all of their time at home, we adjusted for time activity to reduce exposure misclassification. We considered three microenvironments of exposure: inside the apartment, outside the apartment, and school/work/other. We did not include time on highway in our adjustment because only two participants spent time on highways and the amount of time they spent was <1 h per day. *Inside Apartment* was assigned the median indoor PNC obtained from measurements collected in each participant’s apartment. The PNC values assigned for *Outside Apartment* and *School/Work/Other* were obtained using a regression model of PNC developed for areas near I-93 and an area >1 km from I-93 in Somerville [47]. *Outside Apartment* was assigned the annual median PNC on Rt-38 immediately adjacent to the Mystic housing development (40,000 particles/cm^3^), and *School/Work/Other* was assigned the annual median PNC for the area >1 km from I-93 (18,000 particles/cm^3^). 

The results of the time-activity surveys, performed at the end of the 3-week HEPA and 3-week sham periods, were used to estimate the fraction of time, *f*, each participant spent in each microenvironment, *i,* (n = 3 total) during an average 24-h period (average of five work days and two non-work days for employed participants and five week days and two weekend days for unemployed participants). We assumed each participant followed the same 7-day schedule during the three weeks preceding each of the two surveys. We then calculated the time-activity adjusted PNC (TAA-PNC) using Equation (2):

(2) $$\mathrm{TAA}-\mathrm{PNC}=\sum_{i}^{n}{\mathrm{PNC}}_{i}\times f_{i}$$

where *PNC_i_* is the microenvironment-specific PNC estimate. 

TAA-PNC exposure estimates were then analyzed for association with blood pressure and the blood biomarkers. We used the GENMOD procedure to assess the impact on diastolic, systolic and pulse pressure by the exposure allocation (either HEPA or sham accounting for crossover) and TAA-PNC for Lag0, Lag1, Lag2, and MA3. We also assessed the impact on blood biomarkers by exposure allocation (either HEPA or sham accounting for crossover), TAA-PNC for MA14 and MA21, adjusted for baseline levels of the same biomarker. 

## 3. Results

### 3.1. Apartment and Participant Information

Ten apartments were <100 m and the other nine were 101–200 m from I-93 (Table 1). Sixteen apartments had at least one kitchen window partially open during the study regardless of season, and seven had a dining room window open. Five apartments had gas stoves; the remainder had electric stoves. The 20 participants were mostly women (17), born outside the USA (17), non-white (15) and non-Hispanic (14) (Table 1). Eight were Haitian. The mean age was 53.9 years and the median body mass index (BMI) was 31 (range: 20–72). Nine participants reported having completed high school. Nine reported being employed at the time of the study. Of these, two reported exposure to work-related combustion products including motor vehicle exhaust. The employed participants reported not being exposed regularly to asbestos, industrial chemicals, coal or wood combustion emissions, or airborne textile fibers in the workplace. Most participants spent a majority of time home both on weekdays (median = 18, range = 12–24 h) and weekend days (median = 23, range = 15–24 h).

While there were some differences between participants who had HEPA first *vs.* sham first, the groups were largely similar (Table 1). The most common self-reported health problems were high blood pressure (12) and high cholesterol (7). One participant (HEPA first) reported a history of a heart attack. The most common medications participants had in their homes were for hypertension (11) and inflammation (7); anti-inflammatory medication use was much more frequent among the participants assigned to HEPA first. Four participants were former smokers. One participant reported having the flu the week prior to the second visit (t = 21 days), and five reported having a cold, the flu or asthma during the week prior to the third visit (t = 42 days).

### 3.2. Impacts of Filtration on PNC in Participant Homes

**Figure 2:**
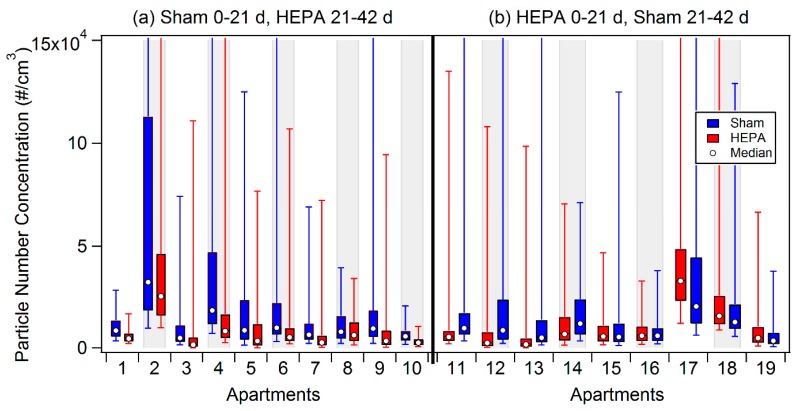
Box plots of all PNC measurements from each participant’s apartment stratified by filter order: (**a**) sham for first 21 days followed by HEPA for last 21 days; (**b**) HEPA for first 21 days followed by sham for last 21 days. The whiskers represent the 5th and 95th percentiles; open circles represent the median PNC values. Note that the apartments are not numbered in chronological order (see Table 2).

In apartments 1–10 (note that the apartment number assignments do not reflect a chronological ordering), where sham filters were used first, consistent particle reductions were observed after changing to HEPA filters (Figure 2). In these apartments the PNC interquartile range was lower and the median PNC decreased on average by 4900 particles/cm^3^ (47%) during the 21 days of HEPA filtration relative to the preceding 21 days of sham filtration (Table 2). In apartments 11–19, where HEPA filters were used first, it was expected that the median PNC would be higher after HEPA filters were switched for sham filters; however, an increase was observed only in apartments 11–14. In these four apartments the median PNC was on average 4700 particles/cm^3^ (52%) higher following the switch to sham filters (Table 2). In apartments 15 and 16 median PNC were largely unchanged after switching to sham filtration, and in apartments 17–19 the median PNC was lower during sham filtration compared to HEPA filtration. The poor removal efficiencies measured in apartments 17–19 could reflect increases in PNC source strength during the sham period relative to the HEPA period. 

**Table 1:** Demographic and health data of the total study population and stratified by intervention order.

Category	Total	HEPA First ^a^	Sham First ^b^
	(*n* = 20)	(*n* = 10)	(*n* = 10)
**Demographic Data**			
Age, Mean (SD), years	53.6 (9.2)	55.6 (11.4)	51.5 (6.3)
BMI, Median (Range)	31.5 (20–72)	30.5 (20–72)	32 (25–51)
Female	16 (80%)	7 (70%)	9 (90%)
Born in USA	4 (20%)	2 (20%)	2 (20%)
White	6 (30%)	2 (20%)	4 (40%)
Hispanic	7 (35%)	3 (30%)	4 (40%)
Household income: <$24,999/year	14 (70%)	8 (80%)	6 (60%)
Household income: $25,000–$74,999/year	3 (15%)	1 (10%)	2 (20%)
Household income: Don’t Know/Refused	3 (15%)	1 (10%)	2 (20%)
High School Diploma or Higher Degree	8 (40%)	4 (40%) **^c^**	4 (40%)
Employed **^d^**	9 (45%)	3 (30%)	6 (60%)
Distance to I-93: ≤100 m	10 (50%)	6 (60%)	4 (40%)
Distance to I-93: 101–200 m	10 (50%)	4 (40%)	6 (60%)
**Health Data and Medicines Used ^e^**			
Total Cholesterol, mean (SD), mg/dL	290 (122)	264 (124)	317 (120)
Triglycerides, mean (SD), mg/dL	211 (141)	169 (111)	254 (160)
Systolic Blood Pressure, mean (SD), mmHg **^f^**	123 (15)	126 (15)	120 (14)
Diastolic Blood Pressure, mean (SD), mmHg **^f^**	77 (10)	80 (10)	75 (9)
Pulse pressure, mean (SD), mmHg **^g^**	45 (10)	46 (11)	45 (9)
Previous Heart Attack	1 (5%)	1 (10%)	0 (0%)
Diabetes	2 (10%)	0 (0%) ^c^	2 (20%)
High Blood Pressure	11 (55%)	8 (80%)	3 (30%)
Anti-hypertension medicine	10 (50%)	7 (70%)	3 (30%)
Anti-inflammatory medicine	7 (35%)	6 (60%)	1 (10%)
Anti-lipids medicine	3 (15%)	2 (20%)	1 (10%)
Anti-diabetes medicine	3 (15%)	1 (10%)	2 (20%)
Former cigarette smoker	4 (20%)	3 (30%)	1 (10%)

Notes: **^a^** HEPA filtration 0–21 days followed by sham filtration 21–42 days; **^b^** Sham filtration 0–21 days followed by HEPA filtration 21–42 days; **^c^** One participant did not answer this question on the survey; **^d^** childcare provider, grocery bagger, cook (2), courier, hotel housekeeper, assistant teacher, eldercare provider, merchandise coordinator; **^e^** The first five parameters in this category were measured for each participant; the remainder were self-reported; **^f^** Average of the right and left arm measurements (from three clinical visits); **^g^** Difference between average systolic and diastolic blood pressure.

**Table 2:** Summary of PNC measurements for each apartment.

Apt. # ^a^	Start Date	Median PNC ^b^			PNC Reduction		Indoor Spikes (%) ^e^	# of Res. ^f^	Notes ^g^
		Sham	HEPA	*p*-Value	% ^c^	Abs. ^d^			
**Sham 0–21 days, HEPA 21–42 days**									
1	11 February 2011	8900	5000	<0.001	44%	3900	0.00	2	K, GS, D_100_
2	25 May 2011	33,000	26,000	<0.001	21%	6900	16	5+	K, GS, AC, D_100_
3	13 July 2011	5300	2100	<0.001	60%	3200	7.5	3	O, K, F, D_200_
4	31 August 2011	19,000	8700	<0.001	54%	10,000	9.0	2	CS, D_100_
5	2 November 2011	9000	3900	<0.001	57%	5200	3.2	2	D_100_
6	19 January 2012	10,000	5700	<0.001	43%	4600	5.4	3	O, DR, K, D_200_
7	10 March 2012	6900	3100	<0.001	55%	3800	2.6	3	K, D_200_
8	4 May 2012	8400	6700	<0.001	20%	1700	1.1	2	O, DR, K, D_200_
9	29 May 2012	9900	3900	<0.001	61%	6000	4.7	4	O, DR, K, AC **^h^**, D_200_
10	21 September 2012	6200	3100	<0.001	50%	3200	0.12	2	K, D_200_
**HEPA 0–21 days, Sham 21–42 days**									
11	11 February 2011	10,000	5800	<0.001	42%	4300	5.5	3	DR, D_100_
12	24 May 2011	9100	2900	<0.001	68%	6100	5.3	5	K, D_100_
13	12 July 2011	5500	2200	<0.001	60%	3300	4.9	5	K, F, D_100_
14	23 September 2011	12,000	7300	<0.001	39%	5000	3.3	3	O, DR, K, AC, D_200_
15	2 November 2011	5900	6000	0.87	No diff.	No diff.	1.8	4	O, DR, K, D_100_
16	19 January 2012	6400	6400	0.53	No diff.	No diff.	0.30	4	K, GS, D_100_
17	13 March 2012	21,000	33,000	<0.001	-	-	11	3	K, GS, D_100_
18	18 July 2012	13,000	16,000	<0.001	-	-	9.3	3	O, K, D_200_
19	14 September 2012	4000	5300	<0.001	-	-	1.8	2	O, DR, K, D_200_

Notes: **^a^** The apartment numbering sequence is arbitrary and not in chronological order; **^b^** Median particle number concentration (particles/cm^3^) based on all measurements made at each apartment; **^c^** Percent reduction in median PNC due to HEPA filtration compared to sham; **^d^** Absolute reduction in median PNC due to HEPA filtration compared to sham; **^e^** Percentage of total PNC measurements >10^5^ particles/cm^3^. Indoor sources likely include cooking and cleaning activities as well as resuspension of settled particles; **^f^** Number of residents living in the apartment during the study; **^g^** O = Another apartment building obstructed the direct pathway between the apartment and I-93; DR = dining room window open during the study; K = kitchen window open during the study; GS = gas stove; AC = air conditioner used at night only; F = floor fan (in LR in apartment 3, in DR in apartment 13); D_100_ = apartment is ≤100 m from I-93; D_200_ = apartment is between 101 and 200 m from I-93; **^h^** AC was only used during days 21–42. Abbreviations: No diff. = no difference in median PNC between HEPA and sham filtration.

PNC reductions due to HEPA filtration were marginally greater (1.0% to 6.6%) in apartments where dining-room windows were kept closed throughout the 42-day intervention (apartments 1-5, 7, 10, 12, and 13) compared to apartments where dining room windows were open for all or part of the intervention period (apartments 6, 8, 9, 11, 14, and 15). The two apartments with the highest overall PNC (2 and 17) had gas stoves, were within 100 m of the highway and had at least one kitchen window open throughout the study. Relationships between PNC and window openness, proximity to I-93 and stove type were not observed for the other apartments.

**Figure A1:**
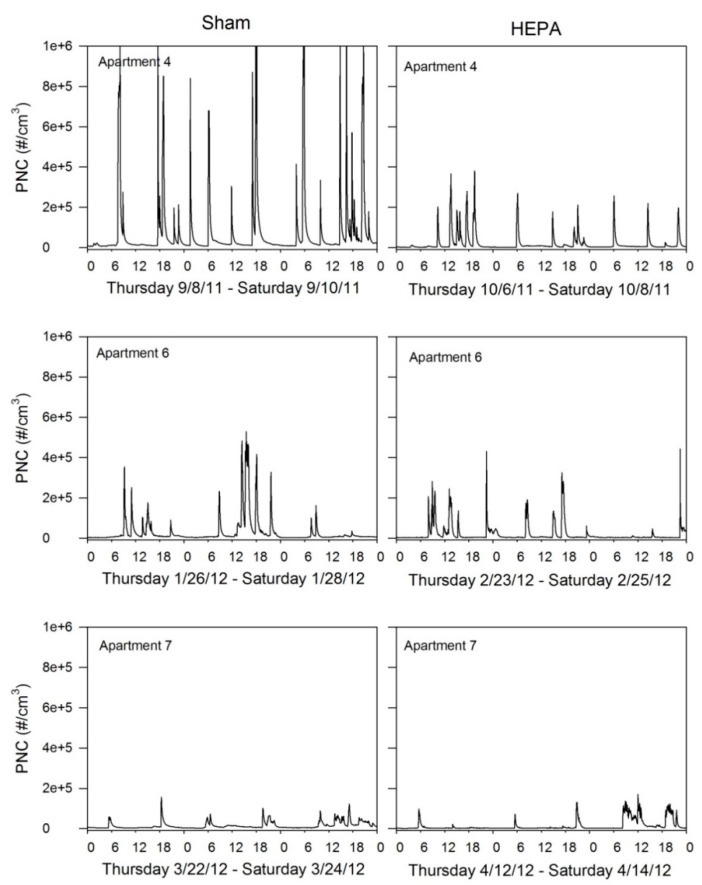
Time-series plots of 1-minute-averaged PNC for three apartments shows the impact of spikes presumably related to indoor activities such as cooking, cleaning, and candle and incense burning.

PNC spikes in excess of 10^5^ particles/cm^3^—the upper limit for discrete-particle quantification by the CPC—were observed in all apartments except Apartment 1 (Table 2). Based on their regular recurrence at approximately the same times of day (Figure A1), we attribute the spikes to indoor activities such as cooking, cleaning activities or candle burning [30]. Incense burning was only observed in one home (#13) during one visit; therefore, it is unlikely that most spikes are due to incense burning. Also, it is unlikely that the spikes are due to cigarette smoking because we specifically recruited non-smoking apartments and we would expect smoking to result in spikes with much shorter recurrence intervals [4]. The percentage of indoor-source spikes (# of 1-min PNC measurements in excess of 10^5^ divided by the total # of 1-min measurements) in the apartments ranged from 0.0% (Apartment 1) to 16% (Apartment 2); the median was 4.7%. The presence of the indoor-source spikes appeared to have had little impact on the PNC reductions attributable to filtration as there were negligible differences between reductions for all hours of the day and for night-time hours only—*i.e.*, when indoor spikes were absent.

### 3.3. HEPA and Sham Filtration Experiments in an Unoccupied Bedroom

**Figure A2:**
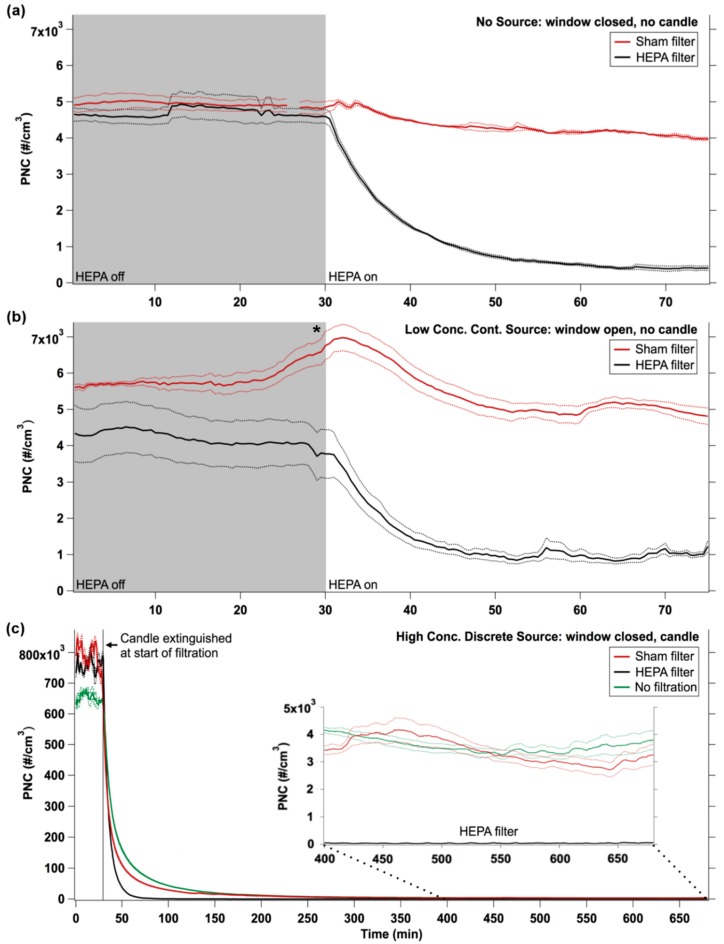
Results of HEPA and sham filtration experiments in an unoccupied bedroom for three PNC-source conditions: (**a**) no source, (**b**) low concentration, continuous source (ambient air through an open window), and (**c**) high concentration, discrete source (a lit candle that was extinguished at t = 30 min (note that HEPA was on at t = 0 min)). Solid lines represent the mean of three replicate experiments; dashed lines represent the standard error (SE). * The increase in PNC was due to an outdoor source that coincided with one of the three repetitions of the experiment.

The results of the HEPA and sham filtration experiments in the unoccupied bedroom (Table A1 and Figure A2) were consistent with our expectations for UFP removal under well-controlled experimental conditions. For the no-source experiments, HEPA filtration lowered PNC 85 ± 3.6% relative to pre-filtration concentrations after 40 minutes (4600 particles/cm^3^ before the HEPA unit was turned on *vs.* 690 particles/cm^3^ after 40 min of filtration). The sham filter lowered PNC only 14 ± 5.8% (4900 particles/cm^3^ before the HEPA unit was turned on *vs.* 4200 particles/cm^3^ after 40 min of filtration). In the experiments with the low concentration, continuous source—an open window—PNC reductions after 40 min of filtration were 74 ± 11% with the HEPA filter (4200 particles/cm^3^ before filtration started *vs.* 1000 particles/cm^3^ after 40 min of filtration) and 12 ± 5.6% with the sham filter (5900 particles/cm^3^ before filtration started *vs.* 5200 particles/cm^3^ after 40 min of filtration). For the high concentration discrete source—a burning candle that was extinguished after the start of filtration (~800,000 particles/cm^3^) and approximated a cooking source—PNC was reduced by 95 ± 0.14% (HEPA) and 84 ± 0.84% (sham) after 40 min of filtration, and after 400 min PNC removals were 99.99 ± 0.0072% for HEPA (measured concentrations were <50 particles/cm^3^) and 99.42 ± 0.13% for sham filtration (measured concentrations were ~3500 particles/cm^3^). Finally, for the high concentration continuous source—a candle that burned throughout the filtration period, PNC levels continued to increase after the start of filtration; however, as expected, the increase was higher during sham filtration compared to HEPA filtration. PNC decreases during sham filtration were likely due to particle losses to stationary surfaces in the room [48].

### 3.4. Blood Biomarkers

As shown in Table 3, we observed no significant differences (paired *t*-test; *p*-value for significance was ≤0.05) in hsCRP, fibrinogen and TNF-RII concentrations in participants after 21 days of exposure to HEPA-filtered air compared with measurements made after 21 days of exposure to sham-filtered air (corrected for baseline and independent of filtration order); however, IL-6 concentrations were statistically significantly higher during HEPA filtration compared to sham. Blood biomarker results stratified by filtration type are shown in Table 4. In the GEE models analyzing the impact of filtration type and order on changes in blood biomarkers, the only statistically significant difference was for change in IL-6 (Table 5), and it was in the direction of a larger increase in IL-6 during the HEPA filtration period. This was true regardless of whether participants were exposed to sham filtration first (apartments 1–10) or HEPA filtration first (apartments 11–19). Our repeated measures analysis produced the same finding. Three participants had high hsCRP (≥10 mg/dL) and two others had very high BMI (>40). Three additional analyses were performed to determine whether these high levels impacted the results: first, the three participants with high hsCRP were removed; second, the two participants with high BMI were removed; and third, all five participants who had either high hsCRP or high BMI were removed. Significant differences between sham and HEPA were found for IL-6 only in the model in which the three participants with high hsCRP were removed (again a decrease in IL-6 during sham exposure). Additional analysis with the GEE model were not possible due to our small sample size. We also examined all blood biomarker values individually across the three sampling times for each participant. As in the prior analysis, IL-6 was the only biomarker for which a trend was seen, but the change was a decrease during sham filtration that was not seen during HEPA filtration (results not shown). 

**Table 3:** Differences in change in blood biomarker and blood pressure measurements due to filtration.

Health Measure	Absolute Difference (95% CI): Sham *vs.* HEPA ^a^	% Difference (95% CI): Sham *vs.* HEPA ^b^
hsCRP (mg/L)	1.05 (0.526–2.11)	5.02 (−45.1–55.1)%
Fibrinogen (mg/dL)	−21.0 (−92.6‒50.6)	64.2 (−154–283)%
TNF-RII (pg/mL)	0.984 (0.862–1.12)	−1.72 (−14.1–10.7)%
IL-6 (pg/mL) *	0.668 (0.465–0.959)	−49.6 (−93.3–−5.90)%
Systolic BP (mm Hg)	8.19 (−0.991‒17.4)	266 (−321–564)%
Diastolic BP (mm Hg)	4.28 (−3.89‒12.4)	367 (−333–1070)%
Pulse Pressure (mm Hg)	3.92 (−4.46‒12.3)	204 (−233–641)%

Notes: **^a^** mean of measurements following 21-day of sham filtration (adjusted for baseline) minus mean of measurements following 21-day of HEPA filtration (adjusted for baseline). CI = confidence interval. hsCPR, TNF-RII, and IL-6 concentrations were first log transformed (natural log) prior to paired *t*-test; the inverse logs were used in the absolute and % difference calculations; **^b^** % difference = ((mean of Sham change—mean of HEPA change)/mean of Sham change) × 100. A % difference >100% indicates a greater reduction in effect during HEPA filtration relative to sham; a % difference >0% and <100% indicates a lesser reduction in effect during HEPA relative to sham; a % difference <0% indicates an increase in effect during HEPA filtration relative to sham. * Significant differences (*i.e.*, *p* ≤ 0.05) between sham and HEPA were observed only for IL-6.

**Table 4:** Blood biomarker concentrations and blood pressure measurements stratified by filter order.

Health Measure	Sham First (0–21 Day), HEPA Second (21–42 Day) (*n* = 10) ^a^			HEPA First (0–21 Day), Sham Second (21–42 Day) (*n* = 10) ^a^		
Day of collection	0	21	42	0	21	42
hsCRP (median (Q1–Q3)) (mg/L)	2.46 (1.75–4.28)	1.45 (1.28–2.29)	1.77 (1.27–2.66)	3.16 (2.16–8.33)	2.97 (0.76–8.17)	5.87 (1.01–11.1)
Fibrinogen (mean ± SD) (mg/dL)	460 ± 104	415 ± 64	410 ± 78	477 ± 132	459 ± 133	439 ± 138
TNF-RII (median (Q1–Q3)) (ng/mL)	2.28 (1.64–2.86)	2.23 (1.92–2.92)	2.28 (1.77–2.42)	2.49 (1.98–2.92)	2.39 (2.10–2.89)	2.14 (1.98–2.70)
IL-6 (median (Q1–Q3)) (pg/mL)	2.10 (1.13–2.89)	1.27 (0.92–1.70)	1.43 (1.10–2.41)	1.58 (1.05–3.32)	1.86 (1.40–3.97)	1.70 (1.28–3.22)
Systolic BP (mean ± SD) (mm Hg)	122 ± 17	120 ± 14	118 ± 12	131 ± 12	121 ± 13	128 ± 18
Diastolic BP (mean ± SD) (mm Hg)	77 ± 10	74 ± 8	73 ± 9	82 ± 9	78 ± 9	81 ± 13
Pulse pressure (mean ± SD) (mm Hg)	45 ± 10	46 ± 11	45 ± 8	49 ± 13	43 ± 12	47 ± 9

Notes: **^a^** number of participants. Abbreviations: Q1 and Q3 are first and third quartile, SD is one standard deviation, BP is blood pressure.

**Table 5:** Percent change from baseline and 95% confidence intervals (CI) for adjusted mean blood biomarker concentrations based on generalized estimation equation model. Means adjusted for baseline blood biomarker concentrations. All variables were analyzed in log scale and transformed prior to reporting.

Blood Biomarker	HEPA 1st (0–21 Day)		Sham 2nd (21–42 Day)		Sham 1st (0–21 Day)		HEPA 2nd (21–42 Day)	
	Adj. Mean Change	CI	Adj. Mean Change	CI	Adj. Mean Change	CI	Adj. Mean Change	CI
hsCRP (mg/L)	−5%	−48%–76%	34%	−26%–144%	−15%	−39%–19%	6%	−10%–25%
Fibrinogen (mg/dL)	−2%	−12%–9%	−5%	−17%–8%	−8%	−17%–1%	−4%	−14%–7%
TNF-RII (pg/mL)	2%	−5%–9%	−6%	−15%–5%	2%	−6%–10%	−2%	−11%–7%
IL-6 * (pg/mL)	24%	9%–40%	−12%	−32%–13%	−24%	−49%–13%	8%	−9%–28%

Notes: Means were adjusted for baseline blood biomarker concentrations. For HEPA 1st and sham 1st, baseline = blood sample collected on day 0; for HEPA 2nd and sham 2nd, baseline = blood sample collected on day 21. All variables were analyzed on the log scale. Values in the table have been converted to normal scale by taking the exponent of the least squared mean for that variable. * Significant differences (*i.e.*, *p* ≤ 0.05) between HEPA 1st and sham 1st were observed only for IL-6.

Tests of TAA-PNC for the lags and MAs are shown in Table 6. TAA-PNC were significantly associated with IL-6 for both MA14 and MA21 (*p* ≤ 0.05) and CRP for MA21 (*p* ≤ 0.05), but the levels of the two biomarkers decreased with increasing TAA-PNC. In contrast, the model with TNF-RII showed a trend toward statistical significance (*p* ≤ 0.10) for MA21 with increasing TAA-PNC associated with a slight increase in TNF-RII.

**Table 6:** Mean change and 95% confidence intervals (CI) in blood biomarker concentrations per 10^4^ particles/cm^3^ increase in PNC moving average (MA) (14- or 21-day MA) adjusted for baseline blood biomarker concentrations.

Biomarker	MA14		MA21	
	exp (β)	CI	exp (β)	CI
hsCRP (mg/L)	−24%	−59–43%	−24% *	−31–−16%
Fibrinogen (mg/dL)	−1%	−6–5%	−1%	−6–5%
TNF-RII (pg/mL)	2%	0–4%	2% **	0–4%
IL-6 (pg/mL)	−19% *	−35–−1%	−18% *	−18–−1%

Notes: * *p* < 0.05; ** *p* < 0.10.

### 3.5. Blood Pressure

In comparing blood pressure measurements from the 20 participants after 21 days of HEPA filtration with measurements made after 21 days of sham filtration, corrected for baseline and independent of filtration order, we observed from paired t-tests that there were no significant differences in systolic, diastolic or pulse pressures (*p*-value for significance was ≤0.05) (Table 3). Blood pressure results stratified by filtration order are shown in Table 4. Systolic, diastolic and pulse pressure were tested for association with TAA-PNC in Lags 0, 1, and 2 and MA3 (Table 7). Pulse pressure in Lag0, Lag2 and MA3 and diastolic in Lag1 were statistically significant (*p* ≤ 0.05) while systolic pressure in Lag1 had a trend toward statistical significance (*p* ≤ 0.10); however, increasing TAA-PNC exposure was associated with decreased blood pressure in all time frames. 

**Table 7:** Mean change and 95% confidence interval (CI) in blood (BP) and pulse pressure (PP) per 10^4^ particles/cm^3^ in PNC (Lag0, Lag1, Lag2, and 3-day moving average (MA3)) after adjustment for baseline blood and pulse pressure, respectively.

BP/PP	Lag0		Lag1		Lag2		MA3	
	β	CI	β	CI	β	CI	β	CI
Systolic BP	0.32	−4.93–5.57	−5.92 **	−12.3–0.51	−4.96	−15.2–5.29	−2.36	−11.4–6.66
Diastolic BP	2.73	−2.66–8.12	−4.45 *	−8.45–−0.45	1.17	−7.16–9.50	2.38	−5.17–9.93
Average PP	−2.88 *	−4.88–−0.88	−2.45	−7.66–2.76	−5.80 *	−9.52–−2.08	−4.36 *	−7.42–−1.30

Notes: * *p* ≤ 0.05; ** *p* ≤ 0.10. BP and PP are in units of mm Hg.

## 4. Discussion

We conducted a small-scale HEPA intervention (n = 20 participants in 19 apartments) in public housing next to a highway in an urban area. Our study is among the first to use HEPA filtration to address infiltration of PNC into homes. Our findings suggest there is a need for more effective filtration to reduce PNC and to increase sample sizes to see biological effects.

### 4.1. PNC Reductions due to HEPA Filtration

PNC reductions achieved by HEPA filtration in the apartments (Figure 2 and Table 2) were much smaller than those achieved in the unoccupied, furnished bedroom (Figure A2 and Table A1). Factors that affected PNC reductions in the apartments included windows being open during the intervention period, room volume, and indoor sources. Participants were asked to keep their windows closed during the 42-day trial; however, we observed during our weekly visits that all but two participants had at least one dining room or kitchen window open during the study (generally the same window). While this tells us that windows were open at least some of the time, we do not know how often windows were open in each apartment. Window opening could have caused PNC increases (infiltration of outdoor particles) or decreases (exfiltration of indoor particles) depending on concentration gradients and air flow patterns. Based on our experience, future HEPA interventions should be designed assuming residents in similar style housing will open their windows.

Room volume was likely a factor in our study. Because the HEPA units were installed in living rooms, which ranged in volume from 35.3 to 40.5 m^3^, the air exchange rate (the number of room-air volumes passing through the filter per unit time) and hence particle removal efficiencies achieved by the HEPA units were reduced compared to our experiments in the unoccupied bedroom, which was 27.8 m^3^ in volume. Importantly, the unoccupied bedroom was well sealed to prevent air exchange with adjacent rooms while the living rooms were not. 

Indoor sources of PNC can include cooking (especially frying meat and oils), burning candles and incense, use of aerosol sprays, and cleaning activities [3]. Based on the pattern of spikes seen in the daily PNC time-series plots for each apartment (see Figure A1), it is evident that indoor sources were common. Despite all of the apartments having vented stove fans, it took minutes to hours for cooking-related PNC spikes to dissipate, similar to what we observed in the unoccupied bedroom experiments (see Figure 2A). It is possible that the increase in median PNC in apartments 17–19 and the lack of a decrease in apartments 15 and 16 after switching from HEPA to sham (Table 2) is attributable to changes in cooking activities in these apartments. It is also possible that changes in kitchen fan usage, window opening, or the number of people in these apartments contributed to the higher than expected levels of PNC during the HEPA period. In addition, the possibility that some participants may have turned off the filters during the intervention cannot be completely ruled out; however, we do not have first-hand evidence based on our weekly visits that this occurred. 

### 4.2. Biological Measurements

There are several possible reasons for not seeing reductions in blood biomarkers levels during HEPA periods or in association with reduced PNC in our study. One was that the period during which participants were exposed to HEPA-filtered air was too short to produce a measurable physiological change. We ran each filter for 21 days, which is longer than some HEPA intervention studies, and within the time needed to see significant changes in blood biomarker levels in observational studies. For example, Hertel *et al.* [16] found CRP associations with PNC for time periods ranging from 2–28 days, with the larger associations at 21 days. Also Delfino *et al.* [11] found associations, some significant, of PNC with CRP, IL-6 and TNF-RII at Lag0 and MA3 and MA9. Our finding of a paradoxical, inverse relationship between sham filtration and TAA-PNC for IL-6 may be due to chance or to unsuccessful randomization for IL-6 levels at baseline.

Another possible reason we did not see reductions in blood biomarker levels could be that the PNC reductions (21%–68%) were too small to impact blood biomarker levels. In a similar study Bräuner *et al.* [37] performed a double-blind crossover trial in which 42 participants (60–75 years old) were exposed for 48 h to HEPA-filtered indoor air and 48 hours to non-filtered indoor air in their homes. They reported average PNC reductions of ~60% but none of the blood biomarkers measured (including CRP, fibrinogen, IL-6 and TNF-RII) showed significant changes following exposure to HEPA-filtered air. In contrast, Allen *et al.* [35] conducted a randomized crossover trial in wood-heated homes where participants were exposed to unfiltered air for 7 days and then to HEPA-filtered air for 7 days. As a result of HEPA filtration, PM_2.5_ levels decreased by 60% and hsCRP levels decreased by 33% on average. Allen *et al.* [35] also reported associations between hsCRP levels and total indoor PM_2.5_ and indoor-generated PM_2.5_, but not with infiltrated PM_2.5_. 

It is also possible that we did not observe changes in blood biomarker levels following HEPA intervention because the majority of the participants in our study (n = 14) were obese (BMI > 31). However, some evidence suggests that obese individuals are more susceptible to the inflammatory effect of traffic-related air pollution [49]. A particular concern, since this was a real-life intervention and not a controlled exposure study, was that participant exposure was likely different than indicated by measurements in the living room. We adjusted for time spent outside the home, but were not able to determine the amount of time residents spent in the living room or the exposure they might have received when they were in others rooms in their apartment. Based on our time-activity data, we know half of the participants reported spending ≥18 h at home during work days and 23 h at home on non-work days. Both time spent in rooms other than the living room and time spent out of the apartment would reduce the potential benefit participants might have received from HEPA filtration.

We also saw no benefit and some inverse associations in which blood pressure declined with increasing PNC levels. Blood pressure has been found to be responsive in short time periods to exposure to particulate air pollution [51] and specifically to PNC [13,15]. Thus, our findings of inverse associations with blood pressure were unlikely to be due to the time window for exposure assessment. Perhaps the lack of a protective effect was due to not achieving sufficient reductions in exposure for the reasons described above. While there are reports of inverse associations of PM and blood pressure in the literature e.g., [50], overall, the evidence for any beneficial health effects of particulate air pollution is slim. Thus, the inverse associations could be due to chance, exposure misclassification or uncontrolled confounding. 

### 4.3. Limitations

Our study had a number of limitations. Our sample size was small, limiting statistical power to see associations. We had a study population that was relatively heterogeneous with a range of co-morbidities and levels of BMI, including two cases of extreme obesity. If these factors are associated with sensitivity or resistance to the effects of UFP, then it is possible that our effective sample size was smaller than 20. In addition, we did not include a washout period in the study design, our time-activity adjustments may have led to underestimation or overestimation of some exposures, and our randomization was not ideal for IL-6 at baseline (higher for sham-first group) or anti-inflammatory use (more frequent for HEPA-first group). Our air pollution data also had limitations. It was restricted to one pollutant, PNC, while the HEPA units would be expected to filter larger particles as well, including PM_2.5_ and PM_10_. However, the sham/HEPA comparison would test for effects from all PM filtration, not only the PNC we measured. We also lacked outdoor measurements of air pollution, but we were able to discern spikes of indoor sources that were probably from cooking and estimate that they contributed only a small (median < 5%) amount to total PNC. 

## 5. Conclusions

We developed, implemented and evaluated a small-scale trial of an intervention designed to reduce traffic-related PNC in public housing near a highway. Reductions in PNC were smaller than we had hoped in most apartments and were actually the same or higher than during sham filtration in some apartments. We found no evidence that the filtration improved blood pressure or blood markers of inflammation or coagulation. However, given the broad evidence for PM affecting cardiovascular health, our small sample size, and the lessons learned from this study, we suggest there is a need for further research that seeks to find effective interventions for near-highway residents. Key elements to future interventions include supplying HEPA filtration in multiple rooms, a larger sample size, and finer grain tracking of resident time-activity patterns.

## Appendix

**Table A1:** Results of filtration experiments in an unoccupied furnished bedroom.

Source	PNC Reduction (%) ^a,b^	
	Sham filter	HEPA filter
None **^c^**	14 ± 5.8	85 ± 3.6
Low concentration, continuous **^d^**	12 ± 5.6	74 ± 11
High concentration		
Discrete (candle, just after being extinguished) **^e^**	83 ± 0.84	95 ± 0.41
Continuous (a burning candle) **^f^**	−32 ± 4.3	−13 ± 7.5

Notes: **^a^** PNC reduction was measured as the difference in PNC averaged over the 30 min prior to HEPA being turned on and PNC averaged over the 10–40 min after HEPA was turned on. For the discrete source, PNC reduction was measured as the difference in PNC averaged over the 30 min prior to HEPA being turned on and the average PNC over the 10–40 min after the source was turned off; **^b^** Mean ± standard deviation (SD), *n* = 3 (except for sham filter with no source where *n* = 2); **^c^** None = no candle; windows closed; **^d^** Window was opened 70 cm (area of opening was 2800 cm^2^) allowing ambient air to enter the room and serve as a continuous source; **^e^** Measurements were performed with HEPA running continuously before and after the candle was extinguished. For the case where the HEPA was turned off (no HEPA or sham filtration), PNC in the room decreased to 77% ± 0.62% after 30 min. Between 400–600 min after the candle was extinguished, PNC reduction due to HEPA and sham filtration were 99.99% ± 0.0072% and 99.42% ± 0.13%, respectively; **^f^** Measurements were performed with candle burning continuously before and after the HEPA was turned on.
